# Relative Virulence of the New Wave

**Published:** 1919-02-15

**Authors:** 


### THE RETURN OF INFLUENZA.

### Relative Virulence of the New Wave.

A FEW days ago there was some doubt whether the recent cases of influenza really indicated a fresh wave, but at the moment there is every sign of a serious, if smaller, epidemic. Deaths from pneumonia are again becoming frequent, and there is a definite rush of hospital cases in most parts of the country.

Undoubtedly, the most common cause of complications following infection is a chill, and the present severe weather, together with the many transport difficulties and industrial unrest, have combined to produce the worst of local conditions. Generally speaking, the disease retains its now classic form, but there are a few minor points of difference in present cases. In the first place, attacks tend to begin rather less suddenly and violently, and the local manifestations in the throat and air sinuses have been distinctly less. Those instances, complicated by pneumonia, tend to follow the usual course of local-consolidation, crisis, and slow clearing of the chest, common in the years before the war. This is in sharp contrast with the rapidly fatal septic type with acute toxalmia characterising the main summer and autumn epidemics, and suggests a distinct decrease in the virulence. It is satisfactory to note that relatively few of those attacked by the main wave have been again infected. Some established immunity would appear to be certain, for most re-infections complain of nothing more than vague rheumatic pains and malaise lasting a few days.

How long this outbreak may be expected to last is difficult to estimate, but the indications are in favour of an early termination if the virulence remains at its present level and the transport arrangements both for people and provisions of the country resume their normal condition. There is no doubt that the correct precautionary means lie ready to hand if adequate nourishment and shelter are within reach. The matter then requires little more than common judgment on the part of the individual in an attempt to raise and maintain his bodily tone.

Recently many investigators have laid the blame of infection upon *Pfeiffer's influenza bacillus*, with the secondary infections by cocci coincident with a lowered bodily resistance. It is interesting to note that on February 11, during the inquest on a man who died suddenly at home, Dr. Spilsbury stated he had found, *post-mortem*, the *Pfeiffer bacillus*, and that death was due to influenzal infection. Such is an acute instance, and in a person aged 52 years, but on the whole it is safe to say that the virulence of the germs is at the moment distinctly lower and suggests the apparent wave as being more strictly due to a lowered public resistance.

